# Phase 1 clinical trial of eneboparatide, a novel PTH receptor 1 agonist

**DOI:** 10.1530/EC-24-0464

**Published:** 2025-06-19

**Authors:** Michel Ovize, Soraya Allas, Michael D Culler, Stephane Milano, Taha Ouldrouis, Mark Sumeray, Jeroen van de Wetering de Rooij, Michael Mannstadt

**Affiliations:** ^1^ AMOLYT Pharma, Ecully, France; ^2^ PRA International, Gröningen, The Netherlands; ^3^ Endocrine Unit, Department of Medicine, Massachusetts General Hospital and Harvard Medical School, Boston, Massachusetts, USA

**Keywords:** hypoparathyroidism, parathormone, calcium, kidney, bone

## Abstract

**Objective:**

This study evaluated the safety, tolerability, pharmacodynamics (PD) and pharmacokinetics (PK) of eneboparatide (AZP-3601), a novel agonist of the PTH receptor 1 developed for the treatment of hypoparathyroidism.

**Design:**

This was a randomized, double-blind, placebo-controlled study. One-hundred four healthy volunteers were recruited into seven single ascending dose (SAD) and five multiple ascending dose (MAD) cohorts.

**Methods:**

PK parameters were time to peak, Cmax, area under the curve (AUC) and half-life. PD parameters included albumin-adjusted serum calcium (sCa), serum phosphorus (sPh), serum endogenous PTH, 24 hr urinary excretion of calcium (24 h-uCa), fractional excretion of calcium (FECa) and bone turnover markers (s-CTX and P1NP).

**Results:**

There were no serious adverse events. All adverse events were of mild-to-moderate intensity. AUC and Cmax of eneboparatide increased with increasing doses. Time to maximum plasma concentration was 5–20 min. SAD showed a dose-dependent increase of sCa and decrease of sPh associated with a reduction of serum endogenous PTH. MAD demonstrated a rapid access to maximal PD effects and maintained levels of sCa throughout the day. Urinary excretion of calcium did not increase as a function of the dose of eneboparatide. P1NP and s-CTX did not change over the treatment period.

**Conclusion:**

The PD effects of eneboparatide were prolonged despite the short half-life. These data suggest that eneboparatide may provide sustained control of serum calcium in patients with hypoparathyroidism with once daily dosing. An open-label phase 2 study in patients with hypoparathyroidism has been recently completed and published and a phase 3 study has been initiated.

**Clinical Trial Registration Number:**

NCT05239221.

## Introduction

Hypoparathyroidism (HP) is a rare disease characterized by the absence or inappropriately low concentrations of circulating parathyroid hormone (PTH), leading to hypocalcaemia and hyperphosphataemia (1, 2, 3, 4).

Clinical presentation of cHP impacts many tissues and organ systems, including the skeletal muscles, brain, heart, and kidneys (5, 6, 7). Standard of care treatment with activated vitamin D and oral calcium does not fully replace the functions of PTH and is associated with long-term complications, such as extra-skeletal calcifications (e.g. kidneys and brain) and cardiovascular diseases (1, 2, 5). Several clinical studies have reported reductions in the quality of life of patients with cHP receiving standard of care treatment (8, 9, 10).

Eneboparatide, also referred to as AZP-3601, is a novel, synthetic, 36-amino acid peptide agonist of human PTH1 receptor, the 1–14 amino acid sequence of which is derived from PTH and the subsequent 15–36 sequence derived from parathyroid hormone-related protein (PTHrP). Eneboparatide has been uniquely designed to potently activate and remain bound to the *R*^0^ conformation of the PTH1 receptor, thereby triggering multiple cycles of G-protein coupling and activation, resulting in a sustained cAMP signal (11, 12).

Nonclinical data in animal models of HP demonstrate that the administration of eneboparatide produces robust and sustained increases in serum calcium without increasing urinary calcium excretion or bone resorption biomarkers (13, 14). The results of a phase 2 open-label study in 28 patients with chronic hypoparathyroidism have recently been reported (15). In this study, 28 patients were enrolled in two consecutive cohorts and were administered daily subcutaneous administration of eneboparatide for 3 months. Eneboparatide was well-tolerated and allowed independence from conventional therapy and maintenance of serum calcium within the target range while normalizing urinary calcium excretion and producing a balanced resumption of bone turnover.

The objectives of this clinical study were to assess the safety and tolerability (primary objective) and pharmacokinetics (PK) and pharmacodynamics (PD) (secondary objectives) of eneboparatide, following single and repeat administration in healthy volunteers.

## Material and methods

### Ethics and privacy

This study was sponsored by AMOLYT Pharma and conducted in a single phase 1 unit (PRA Health Sciences, The Netherlands). The clinical study protocol and informed consent forms were approved by the Independent Ethics Committee of the Foundation ‘Evaluation of Ethics in Biomedical Research’ (Stichting BEBO The Netherlands). The study was conducted in accordance with the Declaration of Helsinki, International Council for Harmonisation E6 (R2) Guideline for Good Clinical Practice (European Medicines Agency/Committee for Medicinal Products for Human Use/ICH/135/1995), the EU Clinical Trial Directive: Directive 2001/20/EC, and the applicable regulatory requirements. All subjects provided written informed consent.

### Trial design

This was a first-in-human, randomized, double-blind, placebo-controlled study in healthy subjects (ClinicalTrials.gov identifier: NCT05239221; EudraCT number: 2020-003295-41). The sponsor designed the protocol and data for dose escalation were reviewed by a Safety Review Committee (SRC). The study consisted of an initial single ascending dose (SAD) part (Part A), followed by a multiple ascending dose (MAD) part (Part B), in which subjects received eneboparatide for 2 weeks. Part B commenced after the first four cohorts of Part A were completed, ensuring there were sufficient available data to enable selection of doses.

### Study population

Healthy males and females of nonchildbearing potential, aged 18–60 years, with a body mass index (BMI) of 19.0–28.0 kg/m^2^ (and body weight ≥50 kg) were eligible.

### Study protocol

Seven sequential cohorts were included in Part A. The first cohort was single-blind and included four subjects, of which three subjects were randomized to receive 5 μg eneboparatide and one subject was randomized to receive placebo. The subsequent cohorts included eight subjects each (six receiving eneboparatide at a dose of 5, 10, 20, 40, 60, 90 and 120 μg, respectively, and two receiving placebo in a randomized and double-blind manner). Subjects in Part A were admitted to the phase 1 study centre on day 1 and received the study drug at the clinical site on day 1 as a single, subcutaneous abdominal injection in the morning. They were discharged on day 4 and were asked to return for a follow-up (FU) visit on day 5–8.

A total of five sequential cohorts were included in Part B with ten subjects in each cohort (eight receiving eneboparatide at a dose of 10, 20, 40, 60 and 80 μg/day, respectively, and two receiving placebo in a randomized and double-blind manner). Subjects in Part B received the study drug (active form or placebo) at the clinical site for 14 consecutive days as daily subcutaneous abdominal injections (with rotation of injection sites every dosing day) in the morning. They were resident in the study unit until day 17 and FU evaluation was performed on day 21–25.

After the injection in Part A and after the last injection in Part B, each subject’s sCa was monitored until values were normal or similar to baseline. Dosing at the next level only began after confirmation of an acceptable safety and PD assessment by the SRC and clearance by the internal ethics committee. The SRC comprised the phase 1 unit principal investigator and medical monitor and the sponsor’s representatives.

### Treatment and blinding

Eneboparatide (NUVISAN GmbH, Germany) was supplied as a lyophilized powder with 40 μg per vial and reconstituted daily with sterile water for injection. Reconstitution volume was 1–2 mL. Volume injected was from 0.25 to 3 mL. The placebo was a saline solution for injection. Individual doses were prepared and labelled at the time of use. A separate randomization list was generated for each part of the study by an independent statistician and kept in a restricted area.

### Safety assessments

Safety and tolerability assessments consisted of adverse events (AEs), clinical laboratory (clinical chemistry, haematology, coagulation, and urinalysis), vital signs (blood pressure, pulse rate, respiratory rate, and temperature), 12-lead ECG (Part B), 24 h continuous cardiac monitoring (telemetry; Part A), body weight, physical examination, injection site reaction, and measurement of antidrug antibodies (ADA) in plasma (Part B).

### Pharmacokinetics assessments

Plasma samples were collected pre-dose and at scheduled time points up to 6 h post-dose on day 1 (Parts A and B) and day 14 (Part B). The analysis of eneboparatide in plasma samples was performed at QPS (The Netherlands) using a hybrid immunoaffinity liquid chromatography–mass spectrometry/mass spectrometry (LC-MS/MS) method, with a validated lower limit of quantification (LLOQ) of 50 pg/mL.

### Pharmacodynamics assessments

Blood samples were taken on day 1 at pre-dose, at several time points post-dose up to day 4 and at FU (Part A), on day 1–14 (pre-dose), and day 15–17 and at FU (Part B) for the analysis of total sCa, albumin-adjusted serum calcium (ADsCa), and sPh. These parameters were also measured on day 1, 7 and 14 at several time points post-dose. Samples for serum creatinine were taken at predefined time points to allow for calculation of fractional excretion of calcium (FECa). Endogenous serum PTH(1–84) was measured on day 1 (pre-dose and 12 h post-dose), day 2–4 and at FU (Part A), on pre-dose on day 1, 7, and 14 and on day 15–17 and FU (Part B). In Part B, additional blood samples were taken on day 1, day 14 and at FU for the analysis of serum bone turnover biomarkers, including carboxy-terminal telopeptide of type I collagen (s-CTx), and procollagen type 1 amino-terminal propeptide (P1NP).

Urine collection was performed over 24 h for the analysis of calcium and creatinine on day 1–3 and at FU (Part A) and day 1, day 7, day 14 and at FU (Part B). FECa (%) was calculated by the formula: FECa = (urine calcium/serum calcium)/(urine creatinine/serum creatinine).

### Statistical analysis

The sample size was determined to obtain adequate safety, tolerability, PK, and PD data while exposing as few subjects as possible to the study treatment. The safety set included all subjects who had received at least one dose of eneboparatide or placebo. The pharmacodynamic set composed all subjects belonging to the safety set and for whom the PD data were considered to be sufficient and interpretable. All individual safety results were listed and descriptive statistics, including change from baseline, were performed, where applicable.

## Results

### Subjects

Fifty-two subjects were included in Part A and received study drug. Thirty-nine subjects received a single dose of eneboparatide and 13 received a single dose of placebo. Fifty-one of 52 subjects completed Part A. One subject was withdrawn from the study on day 3 after receiving 90 μg eneboparatide due to COVID-19. This subject was not replaced. All 52 subjects were included in the safety, PK and PD sets. Baseline characteristics of the Part A population are presented in Table 1. Among the 52 subjects who were included in Part B, 42 subjects received multiple sc doses of eneboparatide and ten received multiple sc doses of placebo. Forty-nine of 52 subjects completed the study. Three subjects did not complete the study due to a withdrawal of consent after the last dose (40 μg eneboparatide), a routine positive SARS-CoV-2 PCR test on day 2 (40 μg eneboparatide) or following moderate AEs including nausea, somnolence, and tremor during the first few days of treatment (60 μg eneboparatide). All 52 subjects were included in the safety set, while 40 and 50 subjects were included in the PK and PD sets, respectively.

**Table 1:** Summary of demographic characteristics (safety set) – Part A and Part B (healthy subjects).

	Part A (SAD) (*n* = 52)	Part B (MAD) (*n* = 52)
Gender (% male)	100%	92%
Age (years; mean (SD)) (min; max)	29 (13) (18–60)	36 (12) (19–60)
BMI (kg/m2; mean (SD)) (min; max)	23 (2) (20–28)	25 (2) (19–28)

### Pharmacokinetics results

**Figure 1:**
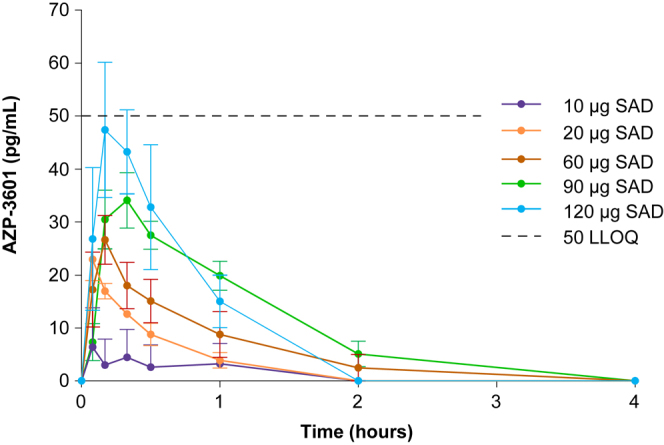
Pharmacokinetics of eneboparatide in SAD. Serum concentration of eneboparatide (in pg/mL) is expressed as a function of time after a single subcutaneous administration at doses (*n* = 6–8/group) ranging from 10 to 120 μg/day. SAD: single ascending dose. Error bars are SEM (standard error of the mean). Eneboparatide was almost undetectable beyond 2 h after administration. The LLOQ was 50 pg/mL.

In this study, most eneboparatide concentrations were below the validated LLOQ of the assay (50 pg/mL). However, concentrations below the LLOQ were detectable and measurable and have been included in the dataset to facilitate the analysis. PK parameters were calculated for subjects where plasma concentrations were available. Plasma concentrations of eneboparatide following a single dose (Part A) appeared to increase in a dose dependent fashion (Fig. 1), consistent with PK linearity. In Part B, the exposure levels and PK profiles of eneboparatide were similar to those measured in Part A for the same dose levels and appeared to be dose-dependent. The average PK profile on day 14 looked similar to the measurements on day 1, suggesting the absence of accumulation of eneboparatide in plasma after 14 consecutive daily administrations of eneboparatide. PK parameters for Parts A and B are shown in Tables 2 and 3, respectively. The median time to maximum plasma concentration was 5–20 min.

**Table 2:** Summary of pharmacokinetic data following single doses of eneboparatide in Part A.

Dose (number of subjects*)	C_max_ (ng/mL) mean ± SD	T_max_ (h) median	AUC_(0–∞)_ (ng·h/mL) mean ± SD
5 μg (*n* = 2)	6.82 ± 2.19	0.25	0.399 ± 0.032
10 μg (*n* = 4)	11.4 ± 4.32	0.22	5.20 ± 3.38
20 μg (*n* = 6)	24.1 ± 8.18	0.10	9.55 ± 4.12
40 μg (*n* = 3)	26.3 ± 4.79	0.08	10.4 ± 13.4 (*n* = 2)
60 μg (*n* = 6)	28.5 ± 10.8	0.17	16.8 ± 14.7
90 μg (*n* = 6)	37.5 ± 11.6	0.33	31.7 ± 12.5
120 μg (*n* = 6)	57.4 ± 28.7	0.33	26.1 ± 16.4

* Pharmacokinetic data are presented for subjects where plasma concentration values were available.

**Table 3:** Summary of pharmacokinetic data following multiple doses of eneboparatide in Part B.

Dose (number of subjects*)	Day	C_max_ (ng/mL) mean ± SD	T_max_ (h) median	AUC_(0–∞)_ (ng·h/mL) mean ± SD
10 μg (*n* = 4)	Day 1	20.6 ± 3.43	0.33	3.26 ± 3.20
10 μg (*n* = 4)	Day 14	18.4 ± 5.37	0.09	2.02 ± 2.41
20 μg (*n* = 8)	Day 1	19.6 ± 5.68	0.13	7.69 ± 5.52
20 μg (*n* = 7)	Day 14	18.3 ± 6.78	0.17	4.63 ± 3.29
40 μg (*n* = 8)	Day 1	23.8 ± 8.97	0.17	15.8 ± 11.5
40 μg (*n* = 8)	Day 14	23.6 ± 5.50	0.17	12.5 ± 4.75
60 μg (*n* = 8)	Day 1	42.9 ± 30.3	0.33	43.3 ± 18.1
60 μg (*n* = 7)	Day 14	44.9 ± 17.9	0.33	58.8 ± 30.3
80 μg (*n* = 8)	Day 1	124 ± 105	0.17	64.2 ± 56.1
80 μg (*n* = 8)	Day 14	63.5 ± 28.3	0.18	51.9 ± 51.6

* Pharmacokinetic data are presented for subjects where plasma concentration values were available.

### Pharmacodynamic results

#### Part A (SAD)

##### Albumin-adjusted serum calcium

**Figure 2:**
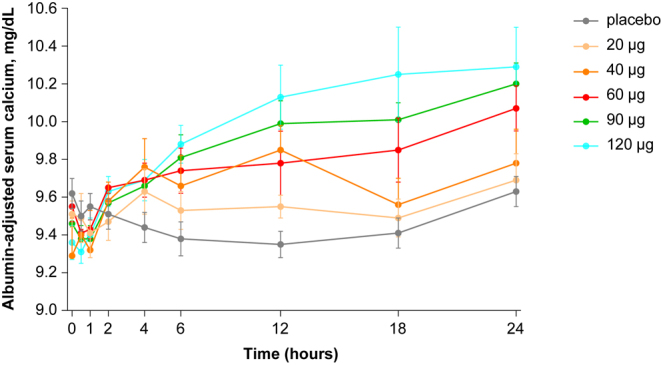
Pharmacodynamics of albumin-adjusted serum calcium in SAD. Albumin-adjusted serum calcium is expressed as a function of time after a single subcutaneous abdominal administration of placebo or eneboparatide at doses ranging from 20 to 120 μg (*n* = 6–8/group). Error bars are SEM. There was an early dose-dependent increase of serum calcium, with values remaining significantly higher than baseline at 24 h for doses of eneboparatide above 40 μg/day.

Moderate variations of ADsCa were observed within 24 h after dosing in the placebo group. They likely correspond to the physiological circadian rhythm, with an initial slow decrease in the morning, a nadir at 12 h post-dose in the afternoon, and an increase to 24 h post-dose (Fig. 2). Administration of 5 and 10 μg eneboparatide blunted this physiological decline in ADsCa during the first 6 h post-dosing. When compared to placebo, administration of 20 μg eneboparatide resulted in a moderate but persistent elevation of ADsCa up to 18 h post-dose (Fig. 2). For the 40, 60, 90, and 120 μg doses of eneboparatide, the rise in ADsCa was more pronounced during the first 4 h and sCa remained elevated up to 24 h with the 40 and 60 μg doses and up to 48 h with the 90 and 120 μg doses of eneboparatide. ADsCa calcium values generally did not exceed the upper limit of the normal range (10.5 mg/dL or 2.6 mmol/L).

##### Serum phosphorus

Administration of 60, 90, or 120 μg eneboparatide reduced sPh levels. When compared to placebo, this reduction was detectable as soon as 2 h after injection, reached a maximum at 6 h, and persisted up to 24 h.

##### Endogenous serum PTH(1–84)

An increase in endogenous sPTH(1–84) was observed in the placebo group at 6 and 12 h post-dose, likely corresponding to the physiological circadian variations of PTH(1–84) secretion. Endogenous sPTH(1–84) levels decreased in a dose-dependent manner following administration of 20–120 μg eneboparatide and persisted for 24–72 h. Supplemental Fig. 1 (see section on Supplementary materials given at the end of the article) depicts the correlation between the increase in sCa after eneboparatide administration and the decrease in endogenous PTH (both measured at 12 h after dosing, *r*^2^ = 0.94).

##### Urine PD

Following administration of increasing single doses of eneboparatide, no apparent dose-related changes in the urinary excretion of calcium or phosphorus were observed, despite a concomitant increase in serum calcium and decrease in serum phosphorus concentrations.

#### Part B (MAD)

##### Albumin-corrected serum calcium

**Figure 3:**
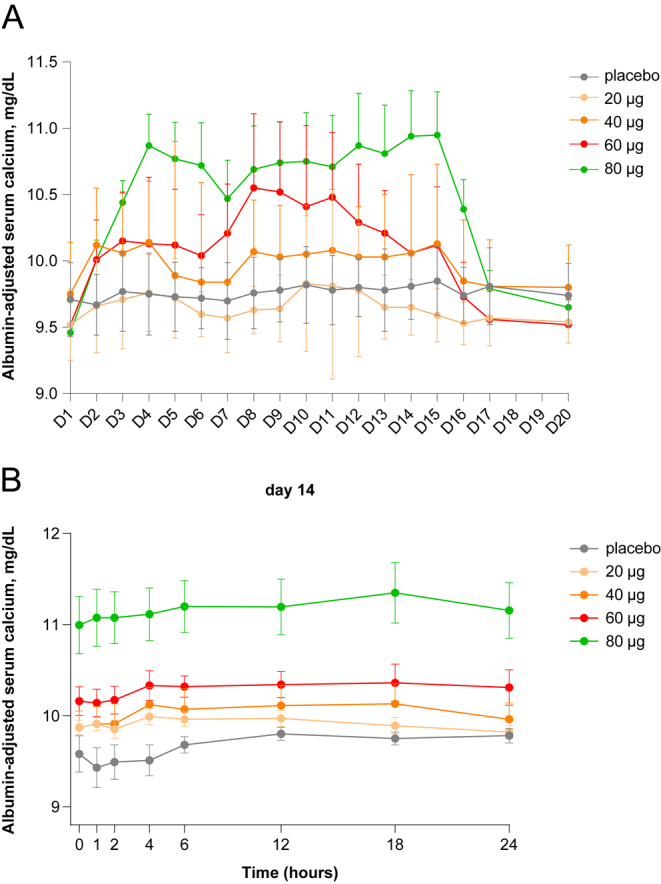
Pharmacodynamics of albumin-adjusted serum calcium in MAD. (A) Mean albumin-adjusted serum calcium during a 14-day treatment period. The mean daily pre-dose albumin-adjusted serum calcium is presented throughout a 14-day treatment period and a subsequent 6 days follow-up phase. Placebo or eneboparatide, at doses ranging from 20 to 120 μg/day, was administered subcutaneously in the abdomen (*n* = 6–8/group). There was a dose-dependent increase of serum calcium, with values remaining significantly higher than baseline from day 2–3 after the start of the treatment up through the end of treatment on day 14. Return to near baseline levels occurred approximately 2–3 days after the last administration of eneboparatide. Error bars are SEM (standard error of the mean). (B) Variations of the mean albumin-adjusted serum calcium during day 14 (last day of treatment). When compared to placebo, administration of eneboparatide induced a dose-dependent increase in the mean albumin-adjusted serum calcium that remained stable throughout 24 h. Error bars are SEM.

Administration of increasing doses of eneboparatide induced a dose-related and sustained increase in morning pre-dosing ADsCa for the duration of the treatment period (Fig. 3A). Stable ADsCa levels were reached approximately 2 days after the first administration for doses up to 60 μg and 4 days for the 80 μg dose that persisted through the remainder of the 14-day treatment period. At 80 μg/d eneboparatide, six of the eight patients displayed transient hypercalcaemia that was well-tolerated and resolved spontaneously after interruption of treatment at day 14. Indeed, urinary excretion of calcium remained normal in five of the six patients with transient hypercalcaemia. After cessation of the treatment, ADsCa levels returned to near baseline levels within approximately 2 days for doses up to 60 μg and 3 days for the 80 μg dose, confirming the long pharmacokinetic effect of eneboparatide. On days 7 and 14, repeated sCa measurements showed stable values with little variation throughout 24 h post-dosing for all doses (Fig. 3B).

##### Serum phosphorus

Following 14 days of treatment with eneboparatide, the mean sPh remained lower in the high-dose groups (above 40 μg/day) compared to placebo for 12–20 h.

##### Endogenous serum PTH

Endogenous sPTH rapidly and dose-dependently declined in the eneboparatide-treated groups compared to placebo, consistent with the observed rise in sCa (Supplemental Fig. 2).

##### Urine PD

**Figure 4:**
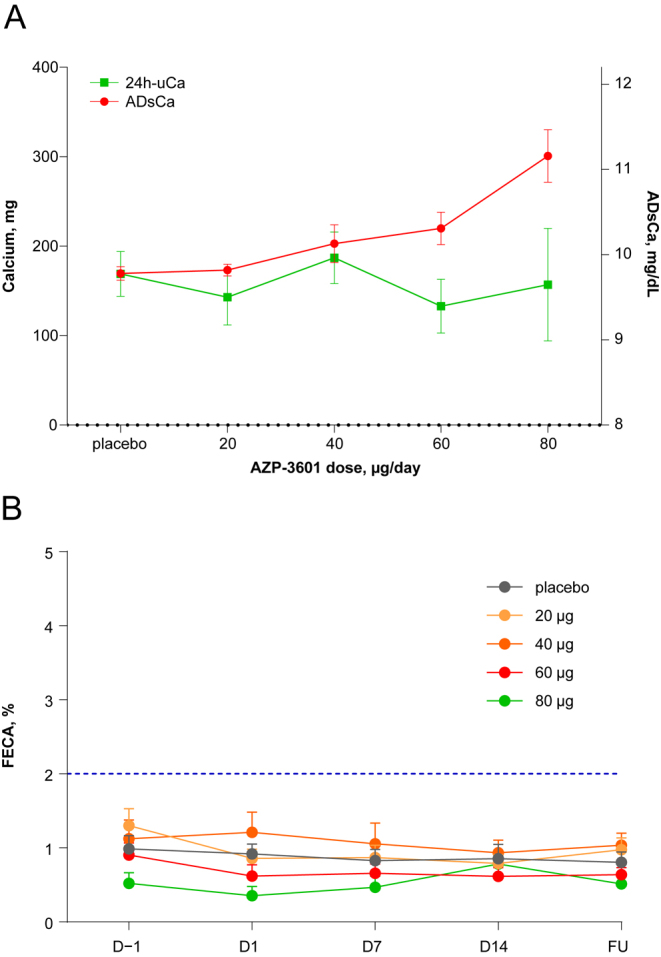
Concomitant effect of increased doses of eneboparatide on serum and urinary calcium. (A) Increasing the dose of eneboparatide resulted in an expected rise in albumin-adjusted serum calcium (ADsCa). Concomitantly, the 24 h urinary excretion of calcium (24 h-uCa) did not significantly increase. Error bars are SEM. (B) FECA was normal (<2%) at baseline. There were no significant changes in FECa whatever the dose of AZP-360. Error bars are SEM.

Similar to healthy volunteers who experienced hypercalcaemia, 14 days of eneboparatide treatment in healthy volunteers was not associated with an increase in the mean 24 h-uCa, despite significant increases in ADsCa (Fig. 4A). FECa remained below 1.5% for all doses of eneboparatide (Fig. 4B).

##### Bone biomarkers

**Figure 5:**
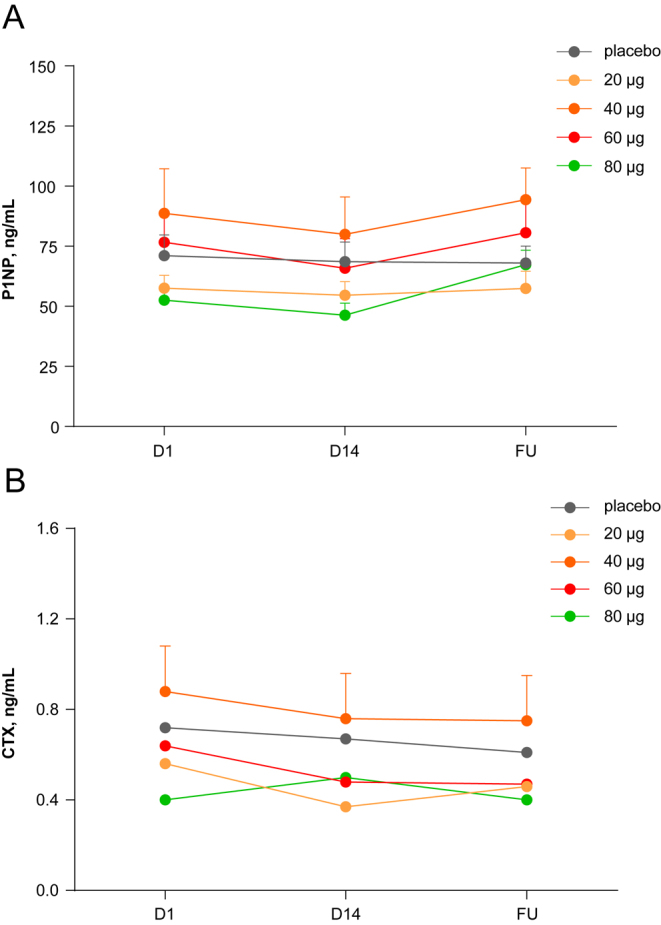
Effect of eneboparatide on markers of bone formation and resorption. Serum levels of P1NP (A) and CTX (B), markers of bone formation and resorption, respectively, were assessed at baseline after 14 days of daily administration of increasing doses of eneboparatide and 5 days after cessation of this treatment (FU: follow-up). Data are presented as changes from baseline values in milligrammes per decilitre (ng/dL). As compared with placebo, eneboparatide did not significantly change the serum levels of either P1NP or CTX. The moderate rise in P1NP levels after stopping the administration of eneboparatide is likely due to the resumption of endogenous PTH release. Error bars are SEM.

Administration of increasing doses of eneboparatide for 14 consecutive days did not alter the serum levels of both P1NP and CTX. A moderate increase in P1NP was observed after cessation of the treatment, likely related to resumption of endogenous PTH secretion (Fig. 5A and B).

### Safety evaluation

Eneboparatide was well-tolerated following single administration up to 120 μg and multiple administration up to 80 μg/day for 14 days. No serious or severe AEs were reported. No changes of clinical relevance were observed for telemetry (Part A) or ECG (Part B), blood pressure, heart rate, or respiratory rate. No relevant changes in safety laboratory values were noted apart from elevated ADsCa values in three out of ten and six out of ten subjects who received 60 and 80 μg, respectively (Part B). At 60 μg, the highest observed calcium result was 11.66 mg/dL on study day 8. At 80 μg, the highest observed calcium result was 13.19 mg/dL on study day 11.

Mild/moderate AEs such as nausea (15 out of 40 subjects), vomiting (four out of 40 subjects), and headache (14 out of 40 subjects) that were associated with hypercalcaemia were reported at the highest doses tested. In Part A, these AEs were transient and observed in eight subjects both for nausea and headache (none for vomiting) at 60, 90, and 120 μg (Table 4). In Part B, these AEs were observed at 40, 60, and 80 μg (Table 5). At 60 μg, these AEs were more pronounced in two subjects; one subject was withdrawn from the study after his second dose. There were no dropouts at 80 μg.

**Table 4:** Treatment-emergent adverse events reported in >5% of subjects in Part A (SAD).

Part A	Total placebo *n* (%)	5 μg	10 μg	20 μg	40 μg	60 μg	90 μg	120 μg	Total eneboparatide *n* (%)
*n*	13 (100)	3	6	6	6	6	6	6	39 (100)
Nausea	1 (7.6)	0	0	0	0	1	3	4	8 (20.5)
Catheter site haematoma	0 (0)	0	1	0	0	0	1	1	3 (7.7)
Fatigue	0 (0)	0	1	1	0	1	0	0	3 (7.7)
Injection site erythema	1 (7.6)	0	0	0	0	0	2	0	2 (5.1)
Injection site pain	4 (13.7)	0	0	1	0	0	2	0	3 (7.7)
Decreased appetite	0 (0)	0	0	1	0	0	2	3	6 (15.4)
Headache	0 (0)	1	1	1	0	0	2	3	8 (20.5)
Somnolence	0 (0)	0	0	0	0	0	1	4	5 (12.8)

**Table 5:** Treatment-emergent adverse events reported in >5% of subjects in Part B (MAD).

Part B	Total placebo *n* (%)	10 μg	20 μg	40 μg	60 μg	80 μg	Total eneboparatide *n* (%)
*n*	10 (100)	8	8	8	8	8	40 (100)
Injection site haematoma	3 (30)	3	3	5	3	3	17 (42.5)
Nausea	3 (30)	0	2	1	5	4	12 (30)
Headache	4 (40)	1	1	3	4	1	10 (25)
Injection site erythema	1 (10)	0	1	4	3	2	11 (27.5)
Somnolence	1 (10)	1	0	3	3	2	9 (22.5)
Catheter site pain	1 (10)	1	0	4	0	1	6 (15)
Catheter site haematoma	1 (10)	3	0	1	0	1	5 (12.5)
Diarrhoea	3 (30)	0	1	2	0	0	3 (7.5)
Injection site pruritus	0 (0)	0	2	3	0	1	6 (15)
Dyspepsia	0 (0)	0	1	2	2	0	5 (12.5)
Myalgia	0 (0)	1	0	4	0	0	5 (12.5)
Flatulence	1 (10)	1	1	1	1	0	4 (10)
Injection site tenderness	1 (10)	0	0	0	2	1	3 (7.5)
Abdominal pain	2 (20)	0	0	1	1	0	2 (5)
Vomiting	0 (0)	0	0	0	3	1	4 (10)
Application site irritation	0 (0)	0	3	0	0	0	3 (7.5)
Fatigue	0 (0)	2	0	0	1	0	3 (7.5)
Decreased appetite	0 (0)	0	0	1	2	0	3 (7.5)
Dry skin	1 (10)	0	2	0	0	0	2 (5)
Injection site pain	0 (0)	1	0	0	2	0	3 (7.5)
Insomnia	0 (0)	0	1	1	1	0	3 (7.5)
Dizziness	0 (0)	0	1	0	1	1	3 (7.5)
Muscle spasms	1 (10)	0	0	2	0	0	2 (5)

Summaries of treatment-emergent AEs reported in >5% of subjects in Parts A and B are provided in Table 3, respectively.

There were no anti-eneboparatide antibodies detected following 14 days of administration with eneboparatide.

## Discussion

This phase 1 study of eneboparatide, a novel PTH/PTHrP hybrid peptide that activates a specific PTH receptor 1 conformation, showed that it was safe and well-tolerated in healthy volunteers following single administration at doses up to 120 μg and repeat administration up to 80 μg/day for 2 weeks. Eneboparatide resulted in a rapid, dose-dependent, and sustained increase in sCa without a concomitant increase in urinary calcium excretion.

It has been demonstrated in animal models that eneboparatide, which has a very short circulating half-life, can produce a greatly prolonged effect on sCa (13, 14). This is related to the specific activation of the *R*^0^ conformation of the PTH receptor 1, as opposed to all other currently available PTH analogues that preferentially activate the R_G_ conformation of the PTH receptor, which is associated with a short duration of action (11). Although some eneboparatide plasma concentration values were below the validated LLOQ of the assay, they were measurable and were reported as they were considered informative. This is expected as eneboparatide is administered at low (microgram) doses and undergoes rapid cellular internalization with prolonged binding to the PTH receptor 1. Therefore, measurement of plasma exposure is challenging due to the very low and transient plasma concentrations achieved.

PK analysis in normal subjects demonstrated that eneboparatide has a less-than-2-hour circulating half-life, in sharp contrast to its very prolonged effect (beyond 24 h) on sCa at doses over 40 μg/day. Indeed, we observed both in the SAD and the MAD studies that while serum eneboparatide concentrations were undetectable, ADsCa was still increasing or was elevated and stable throughout the day.

A second important observation is that, for any dose used in the MAD study, ADsCa remained very stable throughout 24 h. In keeping with this prolonged PD effect, circulating levels of endogenous serum PTH were dramatically decreased in a dose-dependent manner.

The amplitude of the dose-dependent response from 10 to 80 μg/day eneboparatide is expected to translate into relevant pharmacological effects in cHP patients and is roughly comparable to that reported by Karpf *et al.* for a dose range of 12–24 μg/day Transcon-PTH, a formulation of PTH(1–34) that produces a prolonged pharmacokinetic response (15). However, changes in sCa level following initiation or cessation of dosing occur more rapidly with eneboparatide administration.

Hyperphosphataemia enhances the risk of long-term development of cardiovascular diseases that is particularly important in patients with renal insufficiency, but also exists in patients with normal renal function (16). We observed that sPh was significantly reduced at higher doses of eneboparatide. This decrease in sPh was apparent after 14 days of treatment in connection with a reduction in the rate of renal reabsorption of phosphorus. The impact of eneboparatide on sPh was primarily observed within the first 12 h after dosing. Clarke *et al.* previously showed that sPh decreased rapidly and significantly after the onset of PTH(1–84) therapy; however, the decrease in serum phosphorus was not associated with an increased elimination of phosphorus in urine (17).

cHP patients have a higher risk of developing renal diseases or to having progressive alterations in renal function (18, 19, 20, 21, 22). Hypercalciuria exacerbated by standard of care therapy is believed to play a major role in the development of renal diseases in cHP patients. It is yet accepted that the reduction of hypercalciuria constitutes a benefit and is a therapeutic objective (23, 24). In this regard, the MAD study brought two very important findings. First, FECa did not increase and remained within the normal range regardless of the dose of eneboparatide and the resulting increase in sCa. Second, the increase in sCa induced by eneboparatide in our MAD study was not accompanied by an increase in 24 h-uCa of calcium, including when sCa was near the upper limit of normal (Fig. 4).

The administration of PTH as a replacement for the standard of care therapy in adult cHP patients is expected to re-activate bone turnover (3). The half-life, the duration of action, and the way in which PTH is administered have a significant impact on the balance between formation and resorption of HP bone. Intermittent PTH exposure stimulates anabolic osteoblastic bone formation and is indeed used for the treatment of osteoporosis (25). Alternatively, when bone is exposed to PTH in a continuous, non-pulsatile/non-intermittent manner, the process of bone turnover is imbalanced in favour of bone resorption and catabolic demineralization of the bone occurs (26). Our MAD study confirmed that there were no significant changes in the sCa levels of either biomarker after 2 weeks of administration in healthy subjects, suggesting that the balance between bone resorption and formation was preserved and, most importantly, that eneboparatide did not increase bone resorption. This pattern is different from that of other PTH analogs, which induced a significant dose-dependent resorptive effect in healthy volunteers (15). These properties will likely be of importance in deciding on a lifetime treatment for cHP patients who often display co-morbidities that alter bone integrity such as age, menopause, pre-existing osteopenia/osteoporosis, renal failure or diabetes.

In conclusion, this Phase 1 study demonstrated that once daily subcutaneous administration of eneboparatide is safe. Eneboparatide dose-dependently increased sCa for more than 24 h, without increasing urinary excretion of calcium. The PD actions of eneboparatide likely reflect a predominant renal effect with a balanced, non-resorptive impact on bone.

Based on these data, a 3-month open-label phase 2a study in patients with cHP has been designed and completed. Data have been previously published showing that eneboparatide was well-tolerated and allowed independence from conventional therapy and maintenance of serum calcium within the target range while normalizing urinary calcium excretion and producing a balanced resumption of bone turnover (27), consistent with the phase 1 data in healthy volunteers reported here with respect to pharmacodynamic response (i.e. serum calcium and urinary calcium) and effects on bone biomarkers.

## Supplementary materials



## Declaration of interest

M Ovize, S Allas, M D Culler, S Milano, T Ouldrouis and M Sumeray were employees of AMOLYT Pharma. J van de Wetering de Rooij and M Mannstadt declare no conflicts of interest.

## Funding

AMOLYT Pharma was the sponsor of this study.
