# Supplementary material for: Phase 1 clinical trial of eneboparatide, a novel PTH receptor 1 agonist
Source: Endocr Connect. 2025 Jun 19;14(6):e240464. doi: 10.1530/EC-24-0464 (PMC12186298; doi:10.1530/EC-24-0464)
Supplement: Supplementary file 1 [file supplementary_materials.pdf]

Supplemental Material

Supplemental Figure 1: Endogenous serum PTH as a function of albumin-adjusted serum calcium

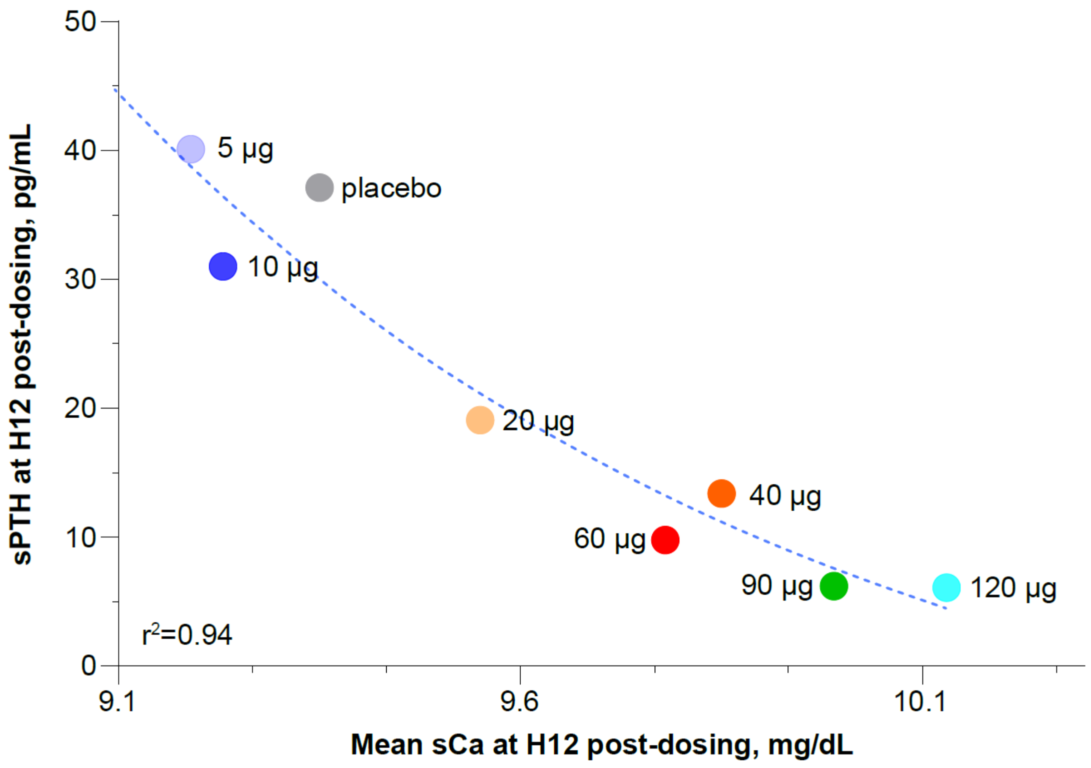

Mean endogenous serum PTH measured at 12 hours (H12) after administration was expressed as a function of mean albumin-adjusted serum calcium. There was a strong relationship between the increase in serum calcium induced by administration of eneboparatide and the reduction of endogenous serum PTH (r^2=0.94).

Supplemental Figure 2: Serum endogenous PTH in MAD

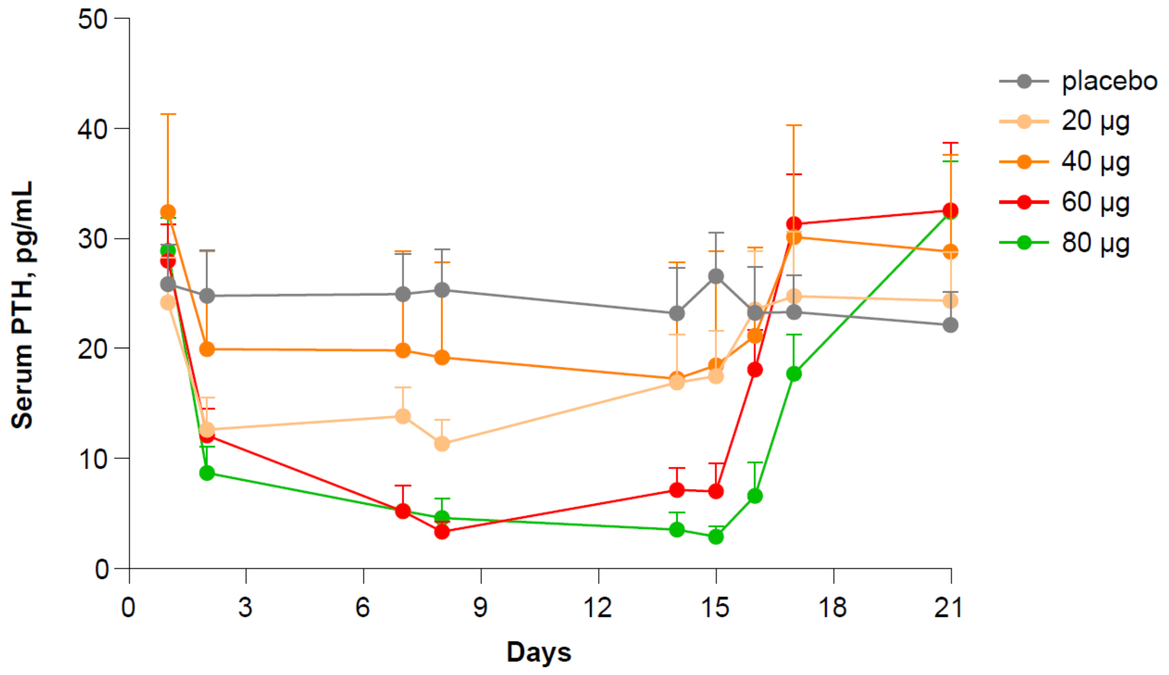

Endogenous serum PTH is expressed as a function of time after throughout a 14-day treatment with increasing doses of eneboparatide ranging from 20 to 80 µg/day of eneboparatide. eneboparatide dose-dependently reduced serum levels of endogenous PTH. This reduction was rapid and persisted throughout the treatment duration. Return to near baseline values occurred at least 2-3 days after stopping eneboparatide administration. Error bars are SEM.
